# DAAs Rapidly Reduce Inflammation but Increase Serum VEGF Level: A Rationale for Tumor Risk during Anti-HCV Treatment

**DOI:** 10.1371/journal.pone.0167934

**Published:** 2016-12-20

**Authors:** Rosanna Villani, Antonio Facciorusso, Francesco Bellanti, Rosanna Tamborra, Annamaria Piscazzi, Matteo Landriscina, Gianluigi Vendemiale, Gaetano Serviddio

**Affiliations:** 1 C.U.R.E. (Centro per la Ricerca e la Cura delle Epatopatie), Institute of Internal Medicine, Department of Medical and Surgical Sciences, University of Foggia, Foggia, Italy; 2 Medical Oncology Unit, Department of Medical and Surgical Sciences, University of Foggia, Foggia, Italy; 3 Laboratory of Pre-Clinical and Translational Research, IRCCS, Referral Cancer Center of Basilicata, Rionero in Vulture, Italy; Chiba University, Graduate School of Medicine, JAPAN

## Abstract

**Background:**

Novel direct-acting antivirals (DAAs) have completely changed the panorama of hepatitis C due to their high efficacy and optimal safety profile. Unfortunately, an unexpectedly high rate of early recurrence of hepatocellular carcinoma has been reported within weeks of starting treatment, but the mechanism is not known.

**Methods:**

We monitored the serum level of vascular endothelial growth factor (VEGF) and changes in the pattern of circulating interleukins in 103 chronic hepatitis C patients during antiviral treatment with DAA-regimens. VEGF, epidermal growth factor (EGF), and several interleukins were assessed at baseline, during treatment, and after treatment. The biological effect of DAA-treated patient serum on human umbilical vein endothelial cell (HUVEC) proliferation was also confirmed.

**Results:**

After 4 weeks of therapy, VEGF increased approximately 4-fold compared to baseline, remained elevated up to the end of treatment, and returned to the pre-treatment level after the end of therapy. In contrast, interleukin-10 and tumor necrosis factor-alpha significantly decreased during therapy, which was coincident with HCV clearance. The levels of both remained low after treatment. The addition of serum from patients collected during therapy induced HUVEC proliferation; however, this disappeared after the end of therapy.

**Conclusions:**

DAA administration induces an early increase in serum VEGF and a change in the inflammatory pattern, coinciding with HCV clearance. This may alter the balance between inflammatory and anti-inflammatory processes and modify the antitumor surveillance of the host. Fortunately, such modifications return reverse to normal after the end of treatment.

## Introduction

The currently available novel combinations of DAAs have completely changed the panorama of hepatitis C therapy. As a result, current HCV infection cure rates have exceeded 90% in a very short time. Data on compensated cirrhosis show rates of sustained virological response (SVR) of around 95–97%, although this is a little lower in more advanced liver diseases [1–5].

High efficacy and an optimal safety profile represent a major breakthrough in the treatment of chronic hepatitis C patients; however, there is still a need to collect further information on the clinical course of patients after viral eradication [6].

In this regard, in a surprising number of patients with hepatitis C and cirrhosis, hepatocellular carcinoma (HCC) seems to develop within weeks of starting treatment with direct-acting antivirals (DAAs) [6, 7].

In a previous study, the authors also included patients with very early HCCs (single tumor <2 cm) or early HCCs with a low to moderate risk of recurrence, in which a complete response was verified using imaging prior to antiviral therapy. These patients showed a probability of recurrence at 4 months of between 17.6 and 21.5%; however, the recurrence rate was approximately 41.2%, which was double the expected rate [6].

Immediately after this study, Conti et al. reported a similar result; however they also observed new diagnoses of HCC [7]. Particularly worrying was the fact that patients who developed HCC after DAAs were younger (median age 56 vs. 73 years) and that, among 16 recurrent cases described by Reig et al., 50% were multinodular and 20% were infiltrative or showed extra-hepatic lesions. In addition, another study observed the occurrence of HCC in advanced liver diseases after DAA administration [8].

As a consequence, the European Medicine Agency (EMA) has extended the scope of its review of the 6 DAAs approved for use in the European Union for treating hepatitis C infection to include the risk of early liver cancer recurrence [9].

On the other hand, a French group analyzed 307 chronic HCV patients with HCC who were treated with DAAs in 3 different cohorts; they concluded that there was no increased risk of HCC recurrence [10].

Before attempting any interpretation of the discrepancies between the above-mentioned reports, it has been postulated that the high rate of tumor relapse after DAA treatment may be dependent on early changes in the microenvironment and viral-induced inflammation in chronic liver injury [11]. There is a fragile balance between the pro- and anti-tumor functions of the immune system. Some studies have proposed that DAA treatment could modify natural killer cell function and expression of the interferon response gene [12, 13]. However, this mechanism is purely speculative and there are no robust preclinical studies to support this hypothesis. The direct pro-oncogenic role of DAA also remains unclear.

The key question is to discover the mechanism responsible for the higher rate of HCC recurrence in patients treated with DAAs. In this regard, Reig et al. proposed that the rapid onset of HCC recurrence upon DAA treatment suggests a sudden increase in cell proliferation without the counterbalance of the immune system. However, they considered it unlikely that DAAs have a direct effect on tumor cell growth, based on the evidence that protease inhibitors reduce the incidence of cancer in HIV patients [6]. They suggested that immune surveillance may be reduced too rapidly as a consequence of the quick decrease in viremia. Even if this is beneficial in terms of liver inflammation, it could be harmful in terms of cancer control.

In Italy, due to budget constraints, the clinical use of DAAs is restricted to patients with more advanced stages of disease, and is based on ethical and clinical considerations. As a consequence, advanced fibrosis and cirrhosis patients with a major risk of HCC development have priority access to antiviral therapy.

We hypothesized that immediately after DAA treatment, the risk of tumor cell dissemination is increased by a drug-induced mechanism. Angiogenesis is a major driver of tumor dissemination, and VEGF is a critical player in liver cancer angiogenesis. Furthermore, VEGF levels in HCC tissues or in circulation correlate with more aggressive disease; therefore, this study was designed to address the hypothesis that increased serum levels of VEGF may be responsible for the higher incidence of HCC recurrence during DAA treatment [14]. Moreover, we also addressed the hypothesis that the change in the circulating inflammatory pattern might occur as a consequence of the reduction in viremia associated with the use of such drugs.

## Patients and Methods

### Study population and study design

We conducted an observational study of 117 patients who started therapy with DAA as standard-of-care treatment for HCV-related liver cirrhosis or advanced fibrosis (METAVIR F3), with or without HCC. All patients were referred to the C.U.R.E. (Centro per la Ricerca e la Cura delle Epatopatie) at the University of Foggia, Italy, between March 2015 and December 2015.

Three patients had a previous diagnosis of HCC and thus, underwent staging and treatment according to the Barcellona Clinic Liver Cancer (BCLC) classification before antiviral therapy. Antiviral therapy was only started after demonstration of a complete response (absence of residual tumor or complete necrosis according to European Association for the Study of Liver Disease criteria). Since patients with active tumors have high serum VEGF level, all of the patients with at least 1 suspected nodule (according to a dynamic pattern or vascular image detected during the arterial phase of dynamic imaging) were excluded from the final analysis.

At baseline and at the end of treatment, all patients were evaluated using abdominal ultrasound. The follow-up of patients with HCCs who achieved a complete radiological response involved contrast-enhanced ultrasound (CEUS) every 3 months and a computed tomography (CT) or magnetic resonance imaging (MRI) scan every 6 months.

All patients with persistent ALT or AST > upper limit of normal (ULN) after 4 weeks of treatment underwent additional US analysis.

Plasma was collected from all patients at multiple time points, including at baseline on the day of starting therapy (T0), after 4 weeks of drug administration (T4wk), on the day of the final treatment (EoT), and at 4 (sustained virological response, SVR4) and 12 (SVR12) weeks post-treatment.

The authors’ institutional review board (UNIFG) approved the clinical investigation, which was conducted according to the principles expressed in the Declaration of Helsinki.

Written informed consent was obtained from all patients to participate in the study.

Only patients with at least 3 serum samples collected at baseline, during treatment (T4wk or EoT), and after the end of therapy (SVR4 or SVR12) were included in the final analysis.

### Hepatitis C treatment

Antiviral therapy and treatment duration (12 or 24 weeks) were indicated for each patient according to the viral genotype/subtype and the severity of liver disease, in accordance with the Italian Association for the Study of Liver Diseases’ (AISF) recommendations and according to the National Drug Agency (AIFA) reimbursement restriction.

Combined treatment based on interferon and DAAs was not included.

Ribavirin (RBV) was administered orally in a divided dose according to body weight (1000 mg daily <75 kg and 1200 mg daily ≥75 kg).

Real-time polymerase chain reaction (PCR) was used for HCV-RNA quantification, with a limit of detection of 12 IU/mL.

Patients were followed up monthly with clinical and laboratory evaluations during antiviral therapy. Virological response was assessed at week 4, at the end of treatment, and at 4 and 12 weeks after the end of treatment to determine the SVR. SVR4 and SVR12 were defined as undetectable HCV RNA 4 or 12 weeks after the treatment completion, respectively.

In patients without a complete 12-week follow-up, the final virological and biochemical status was reported. Failure of DAAs and any adverse events were also recorded.

### Cytokine measurement and biochemical analysis

Cytokine Array I is based on Biochip Array Technology (BAT) by Randox Laboratories, which utilizes multiplex testing methodology to accurately analyze 12 cytokines. The array was used to measure plasma levels of interferon-gamma (IFN-gamma), interleukin-1alpha (IL-1alpha), interleukin-1beta (IL-1beta), interleukin-2 (IL-2), interleukin-4 (IL-4), interleukin-6 (IL-6), interleukin-8 (IL-8), interleukin-10 (IL-10), monocyte chemotactic protein-1 (MCP-1), tumor necrosis factor-alpha (TNF-alpha), and VEGF. The assay was performed at the central laboratory using Randox apparatus, as per the manufacturer’s recommendations using serum from patients taken at each of the follow-up time points. Each sample was tested in duplicate, and the mean was calculated.

Liver function, HCV-RNA, and cytokines were assessed at baseline (T0), after 4 weeks of treatment (T4wk), at the end of treatment (EoT), and 4 and 12 weeks after the end of treatment.

Cytokines were routinely measured in all patients during antiviral therapy.

### Angiogenic activity of serum of DAAs-treated patients

Human umbilical vein endothelial cells (HUVEC) were cultured in HUVEC basal medium (CC-3156 Lonza, Italy) supplemented with an EGM-2 Bullet kit (CC-3156, Lonza, Italy) according to the manufacturer's instructions.

For the HUVEC stimulation assay, cell viability was assessed using the trypan blue test and 5,000 cells were seeded in 24-well plates. After 24 hours, cells were stimulated with:

- complete standard medium (positive control);
- standard medium diluted 1:15 to significantly reduce growth factors and nutrients (negative control);
- 1:15 diluted standard medium supplemented with 100 μl of patient’s serum.

Cells were cultured for a further 72 hours in a humidified atmosphere at 37°C and 5% CO_2_, and were then counted using the Countess II FL Automated Cell Counter (Life Technologies). Three replicates were performed for each experimental condition.

### Statistical analysis

Categorical variables were described as frequencies and percentages. Continuous variables were described as medians and ranges. Log_10_-based transformation of VEGF and IL-8 levels was performed in order to improve normality of distribution and to attain a narrower numeric range to plot.

Temporal trends of cytokines were studied using a non-parametric Wilcoxon signed-rank test and several comparisons were performed between consecutive time points, as well as comparisons between the time points and baseline. Each analysis was conducted for the whole study population and in 2 subgroups that were differentiated according to treatment regimen (sofosbuvir-based vs. ombitasvir+paritaprevir+ritonavir with or without dasabuvir). Comparisons between cytokine levels in the 2 subgroups at each time point were performed using a Kruskal-Wallis test.

Cell culture data are presented as mean and standard deviation of the mean. The difference of the means was calculated using a two-tailed Student's t test.

Statistical analysis was performed using R Statistical Software (Foundation for Statistical Computing, Vienna, Austria) and significance was established when the p value was <0.05 (two-sided).

## Results

### Patients

Table 1 describes the main demographical and clinical characteristics of the population.

**Table 1:** Demographical and clinical characteristics of the population.

Variable	N = 103	
Age (years)	68 (38–84)	
Gender (M/F)	56 (54.4%)/47 (45.6%)	
Genotype	1a	7 (6.8%)
	1b	59 (57.3%)
	2	23 (22.3%)
	3	8 (7.8%)
	4	6 (5.8%)
Treatment Duration 12/24 wks n (%)	62 (60.2%)/41 (39.8%)	
Cirrhosis	52 (50.5%)	
	CHILD-A	46 (44.7%)
	CHILD-B	6 (5.8%)
IL28b Genotype	CC	16 (15.5%)
	CT	35 (34%)
	TT	8 (7.8%)
	na	44 (42.7%)
ALT	<1.5 x ULN	53 (51.5%)
	>1.5 x ULN	50 (48.5%)
Log_10_ HCV RNA (IU/mL)	5.5 (2.3–6.8)	
Treatment Regimen	Sofosbuvir + Ribavirin	28 (27.2%)
	Sofosbuvir + Simeprevir	19 (18.4%)
	Sofosbuvir/Ledipasvir	11 (10.7%)
	Sofosbuvir + Daclatasvir	15 (14.6%)
	Ombitasvir + Paritaprevir + Ritonavir ± Dasabuvir	30 (29.1%)
Use of Ribavirin, n (%)	42 (40.8%)	
Previous treatment	PEG-IFN plus ribavirin	38 (36.9%)
	First generation DAAs	11 (10.7%)
	Second generation DAAs	1 (0.9%)
	Naive	53 (51.5%)

Variables are expressed as an absolute number (percentage) or median (range) when appropriate.

na: not available; ALT, alanine aminotransferase; ULN, upper limit of normal.

Study participants were equally distributed in terms of sex (54.4% men vs. 45.6% women). The *IL28B* genotype was mostly CT (34%) and nearly half of study population (48.5%) had baseline ALT levels >1.5 x ULN. The median baseline log_10_ HCV RNA level was 5.5 IU/mL (range 2.3–6.8 IU/mL) with genotype 1 the most frequently detected (64.1%). After application of exclusion criteria, 103 patients were included in the final analysis; 30 (29.1%) were treated with ombitasvir+paritaprevir+ritonavir with or without dasabuvir and 73 (70.9%) with sofosbuvir (sofosbuvir+simeprevir for 19 patients, sofosbuvir+ribavirin for 28 patients, sofosbuvir+ledipasvir for 11 patients, and sofosbuvir+daclatasvir for 15 patients). All study participants reached SVR at the end of the treatment and 1 relapse was observed after treatment.

**Fig 1:**
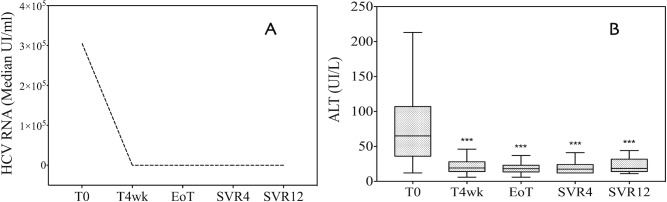
Viral kinetics and liver function test before, during, and after direct antiviral agent (DAA) treatment. (A) HCV viremia during antiviral treatment and after the end of therapy. Within the first 4 weeks of treatment, DAAs induced a rapid decrease in HCV RNA. (B) ALT measurement at baseline (T0), during antiviral treatment (T4wk and EoT), and 4 and 12 weeks after the end of therapy (SVR4 and SVR12). ALT values are shown as the median and interquartile range (IQR). ALT: alanine aminotransferase; ***: p<0.001 vs. baseline.

Treatment with DAAs induced a rapid and early clearance of serum HCV RNA (Fig 1A) that was associated with normalization of ALT (Fig 1B).

#### Growth factors and cytokine kinetics during antiviral treatment

At baseline, no difference in cytokine levels was observed between the 2 treatment regimens, except for IL-8, which was slightly higher in patients treated with ombitasvir+paritaprevir+ritonavir ± dasabuvir (S1 Table).

**Fig 2:**
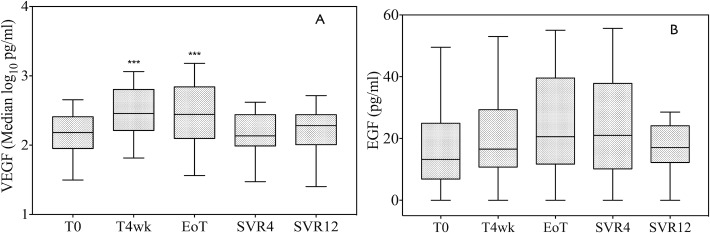
Vascular endothelial growth factor (VEGF) and epidermal growth factor (EGF) serum levels at baseline (T0), during antiviral treatment (T4wk and EoT), and after the end of therapy (SVR4 and SVR12). (A) A significant increase in VEGF serum level was observed after 4 weeks of treatment (p<0.001 vs. T0) and this high concentration persisted up to the end of therapy (p<0.001 vs. T0). At 4 and 12 weeks after the end of treatment, the VEGF serum level reverted to baseline. VEGF level is shown as the median and interquartile range (IQR). Outliers are not reported. (B) EGF values are shown as the median and interquartile range (IQR). Outliers are not reported. No significant differences were observed in EGF level over time. T0: baseline; T4wk: after 4 weeks of therapy; EoT: end of treatment; SVR4: 4 weeks after the end of treatment; SVR12: 12 weeks after the end of treatment; ns = not significant. ***: p<0.001 and *: p≤0.05 vs. baseline.

As shown in Fig 2A, the median log_10_VEGF was significantly increased at 4 weeks after initiation of therapy (from 2.18 log_10_ pg/mL to 2.45 log_10_ pg/mL, p<0.001), remaining higher at the end of treatment (2.44 log_10_ pg/mL, p<0.001 vs. T0), and decreasing to the baseline value upon treatment discontinuation (2.13 log_10_ pg/mL at SVR4 and 2.28 log_10_ pg/mL at SVR12). Of note, VEGF increase was independent from the treatment regimen since it was observed in both therapy groups (S2 Table).

**Fig 3:**
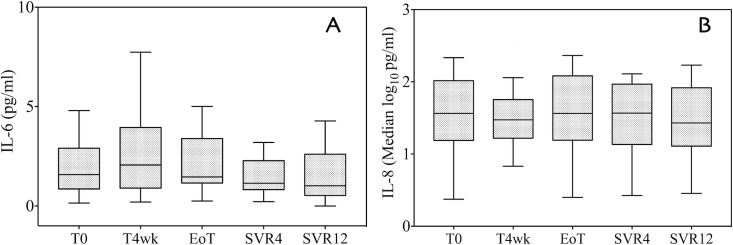
Cytokine measurements before, during, and after DAAs treatment. Interleukin-6 (A) and interleukin-8 (B) serum levels at baseline (T0), during antiviral treatment (T4wk and EoT), and after the end of therapy (SVR4 and SVR12). Values are shown as the median and interquartile range (IQR). Outliers are not reported. T0: baseline; T4wk: after 4 weeks of therapy; EoT: end of treatment; SVR4: 4 weeks after the end of treatment; SVR12: 12 weeks after the end of treatment.

On the other hand, EGF levels also increased during DAA treatment and subsequently decreased at SVR12; however, this trend was not significant (Fig 2B). IL-6 increased from 1.43 pg/mL to 2.78 pg/mL (p = 0.39) at 4 weeks, then steadily declined during therapy, and decreased to an even lower level as compared to baseline at SVR12 (Fig 3A). The trend did not reach statistical significance when considering all patients, nor when treatment subgroups were analyzed (S3 Table).

Log_10_ IL-8 serum levels remained largely stable across time points with respect to baseline levels (Fig 3B and S3 Table).

**Fig 4:**
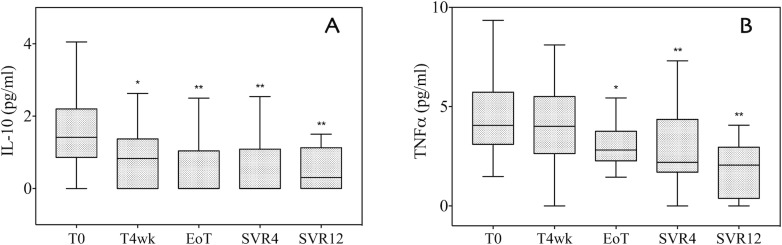
Interleukin-10 (IL-10) and tumor necrosis factor-alpha (TNF-alpha) serum level at baseline (T0), during antiviral treatment (T4wk and EoT), and after the end of therapy (SVR4 and SVR12). (A) IL-10 concentration decreased after 4 weeks of treatment (p = 0.03 vs. T0) and at EoT (p = 0.01 vs. T0); the level of IL-10 remained low up to SVR12. (B) TNF-alpha decreased progressively during treatment and reached statistical significance at the end of treatment (p = 0.05 vs. T0). The median significantly decreased after the end of treatment, at SVR4 and SVR12 (p<0.01). IL-10 and TNF-alpha are shown as the median and interquartile range (IQR). Outliers are not reported. *: p≤0.05 and **: p<0.01 vs. baseline. T0: baseline; T4wk: after 4 weeks of therapy; EoT: end of treatment; SVR4: 4 weeks after the end of treatment; SVR12: 12 weeks after the end of treatment.

As reported in Fig 4, the temporal trends of the 2 main inflammatory cytokines, namely IL-10 and TNF-alpha, were different. At 4 weeks, IL-10 significantly decreased (from 1.44 pg/mL to 0.83 pg/mL; p = 0.03), whereas TNF-alpha levels remained stable (from 4.05 pg/mL to 4.01 pg/mL, p = 0.27) (Fig 4A and 4B, S4 and S5 Tables). Interestingly, while IL-10 levels were substantially suppressed from the end of treatment onwards, the decrease in TNF-alpha started later; it was nearly significant at the end of treatment (2.82 pg/mL, p = 0.05) and this was confirmed, although with a smaller decrease, at each consecutive time point (2.19 pg/mL and 2.09 pg/mL at SVR4 and 12, respectively).

These findings were confirmed in subgroup analysis conducted according to the treatment regimen used (S1–S5 Tables).

### Biological effects of DAA-treated patient serum on endothelial cell growth

**Fig 5:**
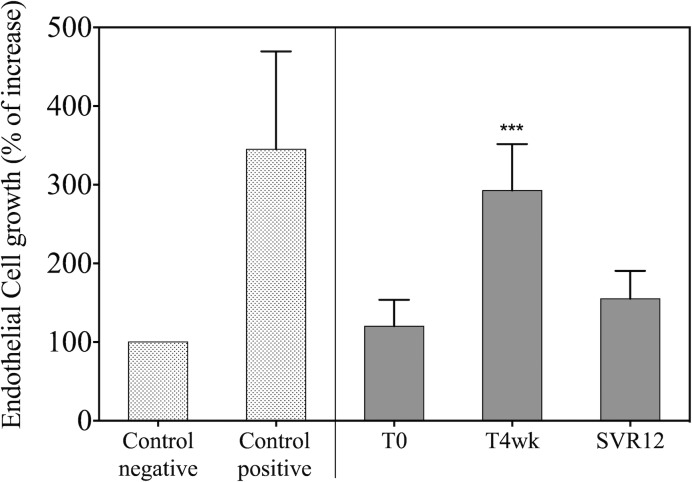
Effects of DAA-treated patient serum on endothelial cell growth. HUVEC proliferation was studied after the addition of serum collected during treatment (T4w) and after treatment (12 weeks after the end of therapy). The high VEGF level achieved during treatment induces a strong biological effect with a dramatic increase in endothelial cell proliferation (T4wk = 290±60%) compared with cellular growth after the addition of serum from the same patients before treatment (T0 = 120±30%). After the end of treatment (SVR12), endothelial cell growth reverted to baseline levels (SVR12 = 160±40%). ***: p<0.001 for T4wk vs. baseline.

Among several tested cytokines, VEGF showed the most significant increase during DAA therapy, as well as a rapid decrease upon treatment withdrawal; therefore, we further evaluated the biological activity of sera collected from patients treated with DAAs using a stimulation assay with human endothelial cells (HUVEC) (Fig 5). HUVEC proliferation was measured upon stimulation with cell medium (diluted 1:15 to reduce standard growth factor and nutrient concentrations) supplemented with sera obtained from patients receiving therapy (T4wk) or at 12 weeks after therapy interruption. This was compared with cell growth under standard culture conditions (positive control). A significant induction of HUVEC proliferation was observed upon supplementation of media with sera from patients receiving DAA therapy (mean: 290±60% vs. 120±30, p<0.0001, Fig 5). Moreover, consistent with the reduction of VEGF upon treatment withdrawal, HUVEC proliferation was comparable to baseline in cell cultures supplemented with sera collected 12 weeks after treatment interruption (SVR12 vs. T0: 160±40% vs. 120±30%, ns). Of note, the peak of HUVEC stimulation upon addition of patient sera was comparable to maximal HUVEC stimulation under standard culture conditions. Taken together, these data suggest that cytokines in the sera of patients receiving DAA are biologically active and able to induce in vitro endothelial cell proliferation.

## Discussion

Hepatitis C virus infection causes significant morbidity and mortality, and affects 150 million people worldwide [15].

European recommendations on the treatment of hepatitis C suggest therapy with DAAs in cirrhotic patients for whom the indication for transplantation is HCC, even though clinical benefits from DAAs have also been suggested in HCC patients without an indication for liver transplantation [16]. However, very recently, Reig et al. questioned the benefits of DAA-based antiviral regimens in such populations, as they observed a significant increase in the early recurrence of tumors [6]. In addition, Conti et al. reported similar results from a retrospective cohort study designed to evaluate the effect of DAA therapy on the development of HCC in HCV-related liver cirrhosis; they found that the proportions of patients who developed HCC after DAA administration were 29% and 3.2% in patients with and without previous HCC, respectively. Moreover, they also raised concerns about the risk of the development of novel HCCs [7].

However, analysis of the results of 3 prospective multicentre cohort studies do not support these conclusions [10]. This last work is particularly relevant since it includes a large number of patients treated with DAAs and focuses on patients previously treated for HCC. It is important to note that in the analysis by Pol et al., only HCC patients treated with curative procedures were considered, while patients treated with transcatheter arterial chemoembolization (TACE) were excluded [10]. In contrast, Reig et al. included all of these patients, who were characterized by high early recurrence rates [6].

The observation of cases with dramatic recurrence/progression within a few weeks of DAA therapy suggests that antiviral therapy may elicit a mechanism favoring tumor growth and dissemination.

In a recent editorial, Nault and Colombo, hypothesized that dysregulation of the anti-tumor response after the considerable decrease in HCV viral load induced by DAA may be the trigger for tumor recurrence [11]. Recurrence could be accelerated when DAAs boost the growth of undetected HCC; this is a consequence of a perturbation of immune surveillance caused by the swift clearance of HCV. The phenomenon is unlikely in patients exposed to IFN owing to the slowly developing antiviral effects of this cytokine coupled with its discrete immune modulatory and anti-proliferative properties [11].

Consistent with this hypothesis, our data show for the first time that during DAA therapy, circulating levels of VEGF increase significantly, remaining high until the end of treatment and declining to the pre-treatment level within 12 weeks of the end of therapy. Of note, an in vitro endothelial cell stimulation assay allowed us to demonstrate that serum from DAA-treated patients has angiogenic properties and is able to significantly induce cell proliferation.

VEGF is considered a critical player in liver cancer angiogenesis, and its elevation in tumor tissue or in circulation correlates with more aggressive disease [14]. VEGF functions as a cytokine, and directly and indirectly affects cancer cell growth and survival; both cancer and endothelial cells depend on VEGF receptor signaling for their survival and function [17]. Indeed, human HCC cells express VEGF and this correlates with the proliferative activity and neoangiogenetic potential of the tumor, probably because VEGF is able to increase endothelial cell proliferation and vessel permeability, and induce disruption of tight junction permeability [18–20]. In a clinical context, elevated circulating VEGF levels after surgery, radiofrequency ablation, or TACE correlate with poor prognosis for patients with HCC and, more interestingly, with a rapid onset of tumor recurrence [14].

In this context, our data provide a strong biological rationale for the surprisingly high number of recurrences of HCC in patients treated with DAAs; moreover, they provide a feasible explanation as to why HCC reappears so early and with rapid clinical dissemination. Indeed, the effect of DAAs seems to be specifically related to VEGF since the serum level of another important growth factor, EGF, remains unchanged (Fig 2B); however, this observation needs to be confirmed by analyzing a larger cohort of patients and other cytokines. Despite this, it is important to note that, as VEGF is a major player in tumor angiogenesis beyond HCC, the risk of cancer dissemination/recurrence may not be restricted to HCC.

Our data also support the hypothesis that the potency of DAA treatment may modify the inflammatory pattern and alter immune surveillance against cancer cells. The effect of DAAs on the host immune response has been recently addressed by Serti et al. who reported the reconstitution of innate immunity after DAA therapy. This was supported by Meissner et al. who showed down-regulation of IFN cell signaling in sofosbuvir+ribavirin regimen therapy [21, 22]. Their observations suggested that the rapid reduction in viremia induced by DAAs may attenuate inflammation with positive implications for hepatitis progression; however, there may be a potentially negative impact on controlling cancer.

Our data confirm that DAAs induce a very rapid decrease in viremia and normalize ALT in less than 4 weeks; together with such improvement, significant decreases in serum IL-10 and TNF-alpha were observed. Rapid reduction in IL-10 and TNF-alpha induced by DAAs depends on the HCV RNA clearance, but it shows a systemic inflammatory pattern change occurring in chronic HCV patients with very long lasting inflammation.

IL-10 is a critical immunoregulatory molecule and its receptors are expressed on the surface of most hematopoietic cells, including T and B cells, and macrophages.

IL-10 is involved in various aspects of the anti-inflammatory process, though its main activity is related to downregulation of T cell function; IL-10 can suppress cytokine production and proliferation of CD4 and CD8 T cells, and can regulate B cell isotype switching and B cell dysregulation. IL-10 may also alter the function of professional antigen presenting cells (APC), such as macrophages and Kuppfer cells [23].

Early blocking of the IL-10 pathway results in rapid viral clearance and suppresses the developing adaptive immune response by altering APC function and exacerbating the dysfunction of T cells [24, 25]. The blunted immune response would favor viral persistence and eventually full exhaustion of virus-specific CD8 T cells [26]. Interestingly, long-term treatment with recombinant IL-10 decreases liver disease in chronic HCV patients, but also leads to increased HCV viral levels via alterations in the host’s immunological viral surveillance [27].

It is clear that the immune system plays a dual role in cancer initiation and progression; inflammatory responses are abundant in tumors and promote their growth, while tumor antigen-specific cytotoxic T cells eliminate tumor cells, but are rare in human tumors. The divergent responses appear to antagonize each other and IL-10 is at the crossroads of this regulation.

Given the observational design of the study, we did not address the possible origin of the VEGF increase; however, our data may provide a rationale for the different results provided by Reig and Pol. In fact, in the study by Pol et al., only patients treated for HCC using curative procedures (resection, percutaneous ablation, or liver transplantation) were considered, whereas Reig et al. also included “non-curative” therapies such as chemoembolization, which is characterized by early recurrence rates [6]. Since we report that DAAs temporarily increase the serum VEGF level, it is conceivable that HCC recurrence only occurs in patients with suboptimal HCC treatment or with residual disease.

We are convinced that such data should be confirmed in a cohort of patients who have received previous treatment for HCC, as well as in large-scale studies. However, we agree with Nault et al., who suggest that “the high rate of HCC recurrence after DAA treatments in patients with prior HCC suggests that a close follow-up of these patients remains mandatory, as well as a reassessment of these observations in prospective dedicated studies” [11].

Fortunately, if our hypothesis is confirmed, the effect of DAAs on inflammation and angiogenesis is limited and reverses soon after the end of treatment.

## Supporting Information

S1 Strobe Checklist The table refers to the Strengthening the Reporting of Observational Studies in Epidemiology (STROBE) statement checklist (information on the STROBE initiative is available at www.strobe-statement.org). (DOC)

S1 Table Temporal trend of IL-8 was studied using a non-parametric Wilcoxon signed-rank test and comparisons were performed between consecutive time points, as well as between the time points and baseline. Each analysis was conducted for the whole study population and in 2 subgroups that were differentiated according to treatment regimen (sofosbuvir vs. ombitasvir+paritaprevir+ritonavir ± dasabuvir).
(DOCX)

S2 Table Temporal trend of Vascular endothelial grow factor (VEGF) was studied using a non-parametric Wilcoxon signed-rank test and comparisons were performed between consecutive time points, as well as between the time points and baseline. Each analysis was conducted for the whole study population and in 2 subgroups that were differentiated according to treatment regimen (sofosbuvir-based vs. ombitasvir+paritaprevir+ritonavir ± dasabuvir).
(DOCX)

S3 Table Temporal trend of IL-6 was studied using a non-parametric Wilcoxon signed-rank test and comparisons were performed between consecutive time points, as well as between the time points and baseline. Each analysis was conducted for the whole study population and in 2 subgroups that were differentiated according to treatment regimen (sofosbuvir-based vs. ombitasvir+paritaprevir+ritonavir ± dasabuvir).
(DOCX)

S4 Table Temporal trend of IL-10 was studied using a non-parametric Wilcoxon signed-rank test and comparisons were performed between consecutive time points, as well as between the time points and baseline. Each analysis was conducted for the whole study population and in 2 subgroups that were differentiated according to treatment regimen (sofosbuvir-based vs. ombitasvir+paritaprevir+ritonavir ± dasabuvir).
(DOCX)

S5 Table Temporal trend of TNFα was studied using a non-parametric Wilcoxon signed-rank test and comparisons were performed between consecutive time points, as well as between the time points and baseline. Each analysis was conducted for the whole study population and in 2 subgroups that were differentiated according to treatment regimen (sofosbuvir-based vs. ombitasvir+paritaprevir+ritonavir ± dasabuvir).
(DOCX)
