# Supplementary material for: DAAs Rapidly Reduce Inflammation but Increase Serum VEGF Level: A Rationale for Tumor Risk during Anti-HCV Treatment
Source: PLoS One. 2016 Dec 20;11(12):e0167934. doi: 10.1371/journal.pone.0167934 (PMC5172554; doi:10.1371/journal.pone.0167934)
Supplement: S1 Strobe Checklist — (DOC) [file pone.0167934.s001.doc]

S1 STROBE Checklist

|  | Item No | Recommendation |
| --- | --- | --- |
| **Title and abstract** | 1 | (*a*) Indicate the study’s design with a commonly used term in the title or the abstract  **Abstract: Page 3 line 8-13** |
|  |  | (*b*) Provide in the abstract an informative and balanced summary of what was done and what was found  **Abstract: Page 3 line 15-23; page 4 line 1-2** |
| Introduction | | |
| Background/rationale | 2 | Explain the scientific background and rationale for the investigation being reported  **Introduction: Page 5 line 2-26; page 6 line 1-24** |
| Objectives | 3 | State specific objectives, including any prespecified hypotheses  **Introduction: page 6 line 25-26; page 7 line 1-7** |
| Methods | | |
| Study design | 4 | Present key elements of study design early in the paper  **Patients and Methods: Study polupation and study design (Page 7)** |
| Setting | 5 | Describe the setting, locations, and relevant dates, including periods of recruitment, exposure, follow-up, and data collection  **Patients and Methods: Study polupation and study design; Hepatitis C treatment; Cytokine measurement and biochemical analysis; Angiogenic activity of serum of DAAs-treated patients (Page 7-10)** |
| Participants | 6 | (*a*) *Cohort study*—Give the eligibility criteria, and the sources and methods of selection of participants. Describe methods of follow-up  **Patients and Methods: Study polupation and study design; Hepatitis C treatment (Page 7-9)**  *Case-control study*—Give the eligibility criteria, and the sources and methods of case ascertainment and control selection. Give the rationale for the choice of cases and controls  *Cross-sectional study*—Give the eligibility criteria, and the sources and methods of selection of participants  **Patients and Methods: Study polupation and study design (Page 7)** |
|  |  | (*b*) *Cohort study*—For matched studies, give matching criteria and number of exposed and unexposed  *Case-control study*—For matched studies, give matching criteria and the number of controls per case |
| Variables | 7 | Clearly define all outcomes, exposures, predictors, potential confounders, and effect modifiers. Give diagnostic criteria, if applicable  **Patients and Methods: Study polupation and study design; Hepatitis C treatment; Cytokine measurement and biochemical analysis; Angiogenic activity of serum of DAAs-treated patients (Page 7-10)** |
| Data sources/ measurement | 8* | For each variable of interest, give sources of data and details of methods of assessment (measurement). Describe comparability of assessment methods if there is more than one group  **Patients and Methods: Cytokine measurement and biochemical analysis; Angiogenic activity of serum of DAAs-treated patients (Page 9-10)** |
| Bias | 9 | Describe any efforts to address potential sources of bias  **Patients and Methods: Study polupation and study design (Page 7)** |
| Study size | 10 | Explain how the study size was arrived at  **Patients and Methods: Study polupation and study design (Page 7)** |
| Quantitative variables | 11 | Explain how quantitative variables were handled in the analyses. If applicable, describe which groupings were chosen and why  **Patients and Methods: Statistical analysis (Page 10); Supplementary Tables 1-5** |
| Statistical methods | 12 | (*a*) Describe all statistical methods, including those used to control for confounding  **Patients and Methods: Statistical analysis (Page 10)** |
|  |  | (*b*) Describe any methods used to examine subgroups and interactions  **Patients and Methods: Statistical analysis (Page 10); Supporting Information S1-5 Tables** |
|  |  | (*c*) Explain how missing data were addressed  **Patients and Methods: Study polupation and study design (Page 7)** |
|  |  | (*d*) *Cohort study*—If applicable, explain how loss to follow-up was addressed  **No patients were lost to follow-up**  *Case-control study*—If applicable, explain how matching of cases and controls was addressed  *Cross-sectional study*—If applicable, describe analytical methods taking account of sampling strategy |
|  |  | (*e*) Describe any sensitivity analyses  **Supporting Information S1-5 Tables** |

Continued on next page

| Results | | |
| --- | --- | --- |
| Participants | 13* | (a) Report numbers of individuals at each stage of study—eg numbers potentially eligible, examined for eligibility, confirmed eligible, included in the study, completing follow-up, and analysed  **Results: Patients (Page 11); Table 1** |
|  |  | (b) Give reasons for non-participation at each stage  **Results: Patients (Page 11); Table 1** |
|  |  | (c) Consider use of a flow diagram  **Table 1** |
| Descriptive data | 14* | (a) Give characteristics of study participants (eg demographic, clinical, social) and information on exposures and potential confounders  **Results: Patients (Page 11); Table 1** |
|  |  | (b) Indicate number of participants with missing data for each variable of interest  **Results: Patients (Page 11); Table 1** |
|  |  | (c) *Cohort study*—Summarise follow-up time (eg, average and total amount)  **Patients and Methods: Cytokine measurement and biochemical analysis (Page 9);**  **Results: Growth factors and cytokines kinetics during antiviral treatment (Page 14-15); Figure 1-4** |
| Outcome data | 15* | *Cohort study*—Report numbers of outcome events or summary measures over time  **Patients and Methods: Cytokine measurement and biochemical analysis (Page 9);**  **Results: Biological effects of DAAs-treated patient serum on endothelial cells growth (Page 17); Figure 1-5; Supporting Information S1-5 Tables** |
|  |  | *Case-control study—*Report numbers in each exposure category, or summary measures of exposure |
|  |  | *Cross-sectional study—*Report numbers of outcome events or summary measures |
| Main results | 16 | (*a*) Give unadjusted estimates and, if applicable, confounder-adjusted estimates and their precision (eg, 95% confidence interval). Make clear which confounders were adjusted for and why they were included  **Supporting Information S1-5 Tables** |
|  |  | (*b*) Report category boundaries when continuous variables were categorized  **Patients and Methods: Statistical analysis (Page 10)** |
|  |  | (*c*) If relevant, consider translating estimates of relative risk into absolute risk for a meaningful time period  **Patients and Methods: Cytokine measurement and biochemical analysis (Page 9);**  **Results: Biological effects of DAAs-treated patient serum on endothelial cells growth (Page 17); Figure 1-5; Supporting Information S1-5 Tables** |
| Other analyses | 17 | Report other analyses done—eg analyses of subgroups and interactions, and sensitivity analyses  **Supporting Information S1-5 Tables** |
| Discussion | | |
| Key results | 18 | Summarise key results with reference to study objectives  **Discussion: page 20 line 8-13; page 20 line 24-26; page 21 line 1-4** |
| Limitations | 19 | Discuss limitations of the study, taking into account sources of potential bias or imprecision. Discuss both direction and magnitude of any potential bias  **Discussion: page 22 line 21-22** |
| Interpretation | 20 | Give a cautious overall interpretation of results considering objectives, limitations, multiplicity of analyses, results from similar studies, and other relevant evidence  **Discussion: page 21 line 7-8** |
| Generalisability | 21 | Discuss the generalisability (external validity) of the study results  **Discussion: page 22 line 18-26** |
| Other information | | |
| Funding | 22 | Give the source of funding and the role of the funders for the present study and, if applicable, for the original study on which the present article is based  **Funding: none** |
