# Supplementary material for: DAAs Rapidly Reduce Inflammation but Increase Serum VEGF Level: A Rationale for Tumor Risk during Anti-HCV Treatment
Source: PLoS One. 2016 Dec 20;11(12):e0167934. doi: 10.1371/journal.pone.0167934 (PMC5172554; doi:10.1371/journal.pone.0167934)
Supplement: S1 Table — Each analysis was conducted for the whole study population and in 2 subgroups that were differentiated according to treatment regimen (sofosbuvir vs. ombitasvir+paritaprevir+ritonavir ± dasabuvir). (DOCX) [file pone.0167934.s002.docx]

|  | Overall (103 pts) | | Sofosbuvir  (73 pts) | | | | Ombitasvir+  Paritaprevir+Ritonavir  ± dasabuvir  (30 pts) | | P^a^ | |
| --- | --- | --- | --- | --- | --- | --- | --- | --- | --- | --- |
| Log_10_VEGF baseline | 2.18 (1.49-2.65) | | 2.17 (1.49-2.65) | | | | 2.18 (1.77-2.62) | | 0.43 | |
| Log_10_VEGF 4 weeks | 2.45 (1.81-3.06) | | 2.46 (1.81-3.06) | | | | 2.34 (1.83-3.06) | | 0.68 | |
| Log_10_VEGF EoT | 2.44 (1.56-3.18) | | 2.62 (1.56-3.18) | | | | 2.29 (1.79-2.84) | | 0.06 | |
| Log_10_VEGF SVR4 | 2.13 (1.46-2.62) | | 2.15 (1.46-2.62) | | | | 2.09 (1.87-2.59) | | 0.23 | |
| Log_10_VEGF SVR12 | 2.28 (1.4-2.71) | | 2.28 (1.4-2.71) | | | | 2.25 (1.92-2.53) | | 0.75 | |
|  | | | | | | | | | | |
|  | Overall | P^b^ | | Sofosbuvir | | P^b^ | | Ombitasvir+  Paritaprevir+Ritonavir ± dasabuvir | | P^b^ |
| Δ Log_10_VEGF w4-0 | 0.27 (0.06-0.48) | **0.001** | 0.29 (0.14-0.48) | | **<0.001** | | | 0.16 (0.06-0.38) | | **0.01** |
| Δ Log_10_VEGF EoT-w0 | 0.26 (0.07-0.53) | **0.001** | 0.45 (0.07-0.53) | | **0.001** | | | -0.09 (-0.1-0.22) | | **0.05** |
| Δ Log_10_VEGF SVR4-w0 | -0.05 (-0.1-0.21) | 0.93 | -0.02 (-0.1-0.03) | | 0.70 | | | -0.09 (-0.13-0.21) | | 0.83 |
| Δ Log_10_VEGF SVR12-w0 | 0.1 (-0.16-0.19) | 0.46 | 0.04 (-0.16-0.19) | | 0.72 | | | 0.07 (-0.09-0.15) | | 0.66 |
| Δ Log_10_VEGF EoT-w4 | -0.01 (-0.25-0.2) | 0.67 | 0.16 (-0.25-0.21) | | 0.28 | | | -0.25 (-0.34-0.04) | | 0.14 |
| Δ Log_10_VEGF SVR4-EoT | -0.31 (-0.56-0.1) | **0.004** | -0.47 (-0.56-0.1) | | **0.004** | | | 0 (-0.25-0.12) | | 0.49 |
| Δ Log_10_VEGF SVR12-SVR4 | 0.15 (-0.06-0.2) | 0.32 | 0.13 (-0.06-0.19) | | 0.25 | | | 0.16 (-0.06-0.24) | | 0.62 |
