# Supplementary material for: DAAs Rapidly Reduce Inflammation but Increase Serum VEGF Level: A Rationale for Tumor Risk during Anti-HCV Treatment
Source: PLoS One. 2016 Dec 20;11(12):e0167934. doi: 10.1371/journal.pone.0167934 (PMC5172554; doi:10.1371/journal.pone.0167934)
Supplement: S2 Table — Each analysis was conducted for the whole study population and in 2 subgroups that were differentiated according to treatment regimen (sofosbuvir-based vs. ombitasvir+paritaprevir+ritonavir ± dasabuvir). (DOCX) [file pone.0167934.s003.docx]

|  | Overall (103 pts) | | Sofosbuvir (73 pts) | | | | Ombitasvir+  Paritaprevir+Ritonavir  ± dasabuvir  (30 pts) | | P^a^ | |
| --- | --- | --- | --- | --- | --- | --- | --- | --- | --- | --- |
| IL-6 baseline | 1.43 (0.15-20.5) | | 1.39 (0.15-20.5) | | | | 1.85 (0.27-12.2) | | 0.95 | |
| IL-6 4 weeks | 2.78 (0.2-46.1) | | 2.42 (0.2-46.1) | | | | 3.34 (0.47-12.51) | | 0.44 | |
| IL-6 EoT | 1.8 (0.25-25) | | 1.44 (0.3-25) | | | | 2.13 (0.25-12.4) | | 0.39 | |
| IL-6 SVR4 | 1.65 (0.22-110) | | 1.61 (0.22-110) | | | | 2.35 (0.93-7.45) | | 0.45 | |
| IL-6 SVR12 | 1.02 (0-11) | | 0.9 (0-11) | | | | 1.75 (1.34-3.15) | | 0.76 | |
|  | | | | | | | | | | |
|  | Overall | P^b^ | | Sofosbuvir | | P^b^ | | Ombitasvir+  Paritaprevir+Ritonavir ± dasabuvir | | P^b^ |
| Δ IL-6 w4-0 | 1.34 (0.05-25.6) | 0.39 | 1.03 (0.05-25.6) | | 0.08 | | | 1.49 (0.2-2.13) | | 0.3 |
| Δ IL-6 EoT-w0 | 0.37 (0.1-4.5) | 0.39 | 0.15 (0.06-4.47) | | 0.27 | | | 0.36 (-0.01-0.74) | | 0.78 |
| Δ IL-6 SVR4-w0 | 0.22 (0.07-89.5) | 0.46 | 0.22 (0.07-89.5) | | 0.36 | | | 1.5 (-4.7-2.72) | | 0.28 |
| Δ IL-6 SVR12-w0 | -0.41 (-8.5-0.1) | 0.28 | -0.51 (-8.5-0.1) | | 0.24 | | | -0.1 (-8.9-0.1) | | 0.42 |
| Δ IL-6 EoT-w4 | -0.98 (-21.1-0.1) | 0.43 | -0.98 (-21.1-0.1) | | 0.49 | | | -1.21 (-2.13-0.22) | | 0.55 |
| Δ IL-6 SVR4-EoT | -0.15 (-0.3-85) | 0.39 | 0.17 (-0.3-85) | | 0.46 | | | 0.18 (-4.95-0.68) | | 0.31 |
| Δ IL-6 SVR12-SVR4 | -0.63 (-99-0) | 0.44 | -0.71 (-99-0) | | 0.48 | | | -0.6 (-4.3-0.41) | | 0.37 |
