# Supplementary material for: DAAs Rapidly Reduce Inflammation but Increase Serum VEGF Level: A Rationale for Tumor Risk during Anti-HCV Treatment
Source: PLoS One. 2016 Dec 20;11(12):e0167934. doi: 10.1371/journal.pone.0167934 (PMC5172554; doi:10.1371/journal.pone.0167934)
Supplement: S3 Table — Each analysis was conducted for the whole study population and in 2 subgroups that were differentiated according to treatment regimen (sofosbuvir-based vs. ombitasvir+paritaprevir+ritonavir ± dasabuvir). (DOCX) [file pone.0167934.s004.docx]

|  | Overall (103 pts) | | Sofosbuvir (73 pts) | | | | Ombitasvir+  Paritaprevir+Ritonavir ± dasabuvir  (30 pts) | | | P^a^ | |
| --- | --- | --- | --- | --- | --- | --- | --- | --- | --- | --- | --- |
| Log_10_IL-8 baseline | 1.5 (0-2.5) | | 1.4 (0-2.5) | | | | 1.8 (1.32-2.5) | | | 0.06 | |
| Log_10_IL-8 4 weeks | 2 (0-3) | | 1.54 (0-3) | | | | 1.53 (1.43-2.41) | | | 0.19 | |
| Log_10_IL-8 EoT | 1.51 (0.39-2.77) | | 1.8 (0.39-2.77) | | | | 1.37 (1.14-2.08) | | | 0.09 | |
| Log_10_IL-8 SVR4 | 1.55 (0.42-3.26) | | 1.60 (0.42-3.26) | | | | 1.24 (0.83-2.13) | | | 0.28 | |
| Log_10_IL-8 SVR12 | 1 (0-2) | | 1.39 (0-2) | | | | 1.38 (0.98-1.88) | | | 0.47 | |
|  | | | | | | | | | | | |
|  | Overall | P^b^ | | Sofosbuvir | | P^b^ | | | Ombitasvir+  Paritaprevir+Ritonavir ± dasabuvir | | P^b^ |
| Δ Log_10_IL-8 w4-0 | 0.5 (0-0.5) | 0.48 | 0.14 (0-0.5) | | 0.24 | | | -0.27 (-0.3-0.13) | | | 0.08 |
| Δ Log_10_IL-8 EoT-w0 | 0.01 (0-0.39) | 0.98 | 0.4 (0-0.39) | | 0.23 | | | -0.43 (-0.5-0.1) | | | 0.21 |
| Δ Log_10_IL-8 SVR4-w0 | 0.05 (0.01-1.76) | 0.75 | 0.2 (0.01-1.76) | | 0.35 | | | -0.56 (-0.65-0) | | | 0.08 |
| Δ Log_10_IL-8 SVR12-w0 | -0.5 (-0.5-0) | 0.81 | -0.5 (-0.5-0) | | 0.83 | | | -0.42 (-0.68-0.34) | | | 0.78 |
| Δ Log_10_IL-8 EoT-w4 | -0.49 (-0.7-0) | 0.49 | 0.26 (-0.7-0) | | 0.25 | | | -0.16 (-0.33-0.1) | | | 0.38 |
| Δ Log_10_IL-8 SVR4-EoT | 0.04 (-0.3-0.49) | 0.63 | -0.2 (-0.3-0.49) | | 0.56 | | | -0.07 (-0.27-0.05) | | | 0.41 |
| Δ Log_10_IL-8 SVR12-SVR4 | -0.45 (-1.2-0.55) | 0.86 | -0.21 (-1.2-0) | | 0.53 | | | 0.14 (-0.25-0.10) | | | 0.67 |
