# Supplementary material for: DAAs Rapidly Reduce Inflammation but Increase Serum VEGF Level: A Rationale for Tumor Risk during Anti-HCV Treatment
Source: PLoS One. 2016 Dec 20;11(12):e0167934. doi: 10.1371/journal.pone.0167934 (PMC5172554; doi:10.1371/journal.pone.0167934)
Supplement: S4 Table — Each analysis was conducted for the whole study population and in 2 subgroups that were differentiated according to treatment regimen (sofosbuvir-based vs. ombitasvir+paritaprevir+ritonavir ± dasabuvir). (DOCX) [file pone.0167934.s005.docx]

|  | Overall (103 pts) | | Sofosbuvir (73 pts) | | | | Ombitasvir+  Paritaprevir+Ritonavir ± dasabuvir  (30 pts) | | | P^a^ | |
| --- | --- | --- | --- | --- | --- | --- | --- | --- | --- | --- | --- |
| IL-10 baseline | 1.44 (0.19-14) | | 1.29(0.19-14) | | | | 1.39 (0.3-4.75) | | | 0.78 | |
| IL-10 4 weeks | 0.83 (0-7.52) | | 0.83 (0-7.52) | | | | 1.26 (0-3.51) | | | 0.47 | |
| IL-10 EoT | 0 (0-2.5) | | 0 (0-2.5) | | | | 0 (0-1.26) | | | 0.63 | |
| IL-10 SVR4 | 0 (0-2.54) | | 0 (0-2.54) | | | | 0 (0-1.23) | | | 0.45 | |
| IL-10 SVR12 | 0.3 (0-1.5) | | 0.3 (0-1.5) | | | | 1.29 (0-1.45) | | | 0.23 | |
|  | | | | | | | | | | | |
|  | Overall | P^b^ | | Sofosbuvir | | P^b^ | | | Ombitasvir+  Paritaprevir+Ritonavir ± dasabuvir | | P^b^ |
| Δ IL-10 w4-0 | -0.61 (-6.5-0.2) | **0.03** | -0.46 (-6.5-0.2) | | **0.05** | | | -0.13 (-1.24-0) | | | 0.08 |
| Δ IL-10 EoT-w0 | -1.44 (-11.5-0) | **0.002** | -1.29 (-11.5-0) | | **0.03** | | | -1.39 (-3.49-0) | | | **0.01** |
| Δ IL-10 SVR4-w0 | -1.44 (-11.5-0) | **0.002** | -1.29 (-11.5-0) | | **0.03** | | | -1.39 (-3.4-0) | | | **0.01** |
| Δ IL-10 SVR12-w0 | -1.41 (-12.5-0) | **0.003** | -1.25 (-12.5-0) | | **0.04** | | | -0.1 (-3.3-0) | | | 0.53 |
| Δ IL-10 EoT-w4 | -0.83 (-5-0) | 0.18 | -0.83 (-5-0) | | 0.18 | | | -1.26 (-2.4-0) | | | 0.09 |
| Δ IL-10 SVR4-EoT | 0 (-0.04-0) | 0.62 | 0 (-0.04-0) | | 0.65 | | | 0 (-0.03-0) | | | 0.55 |
| Δ IL-10 SVR12-SVR4 | 0.3 (-1-0.5) | 0.45 | 0.3 (-1-0.5) | | 0.48 | | | 1.29 (0-1.29) | | | 0.10 |
