# Supplementary material for: DAAs Rapidly Reduce Inflammation but Increase Serum VEGF Level: A Rationale for Tumor Risk during Anti-HCV Treatment
Source: PLoS One. 2016 Dec 20;11(12):e0167934. doi: 10.1371/journal.pone.0167934 (PMC5172554; doi:10.1371/journal.pone.0167934)
Supplement: S5 Table — Each analysis was conducted for the whole study population and in 2 subgroups that were differentiated according to treatment regimen (sofosbuvir-based vs. ombitasvir+paritaprevir+ritonavir ± dasabuvir). (DOCX) [file pone.0167934.s006.docx]

|  | Overall (103 pts) | | Sofosbuvir (73 pts) | | | | Ombitasvir+  Paritaprevir+Ritonavir ± dasabuvir  (30 pts) | | | P^a^ | |
| --- | --- | --- | --- | --- | --- | --- | --- | --- | --- | --- | --- |
| TNFα baseline | 4.05 (1.48-12.01) | | 3.7 (1.48-12.01) | | | | 5.23 (2.17-10.6) | | | 0.28 | |
| TNFα 4 weeks | 4.01 (0-13.07) | | 4.16 (0-13.07) | | | | 5.16 (1.65-7.55) | | | 0.82 | |
| TNFα EoT | 2.82 (0-6.8) | | 2.85 (0-6.8) | | | | 4.26 (0-6.4) | | | 0.21 | |
| TNFα SVR4 | 2.19 (0-7.31) | | 2.37 (0-7.31) | | | | 3.64 (0-5.7) | | | 0.42 | |
| TNFα SVR12 | 2.09 (0-7) | | 2.11 (0-7) | | | | 1.79 (0.89-3.14) | | | 0.26 | |
|  | | | | | | | | | | | |
|  | Overall | P^b^ | | Sofosbuvir | | P^b^ | | | Ombitasvir+  Paritaprevir+Ritonavir ± dasabuvir | | P^b^ |
| Δ TNFα w4-0 | -0.04 (-1.48-0.2) | 0.27 | 0.34 (-1.48-0.2) | | 0.11 | | | -0.08 (0.52-0) | | | 0.52 |
| Δ TNFα EoT-w0 | -1.23 (-3.21-0.3) | **0.05** | -0.85 (-3.21-0.3) | | 0.09 | | | -0.58 (-3.1-0.2) | | | 0.13 |
| Δ TNFα SVR4-w0 | -1.86 (-4.7-0.2) | **0.007** | -1.33 (-4.7-0.2) | | **0.04** | | | -1.61 (-4.5-0) | | | **0.01** |
| Δ TNFα SVR12-w0 | -1.96 (-5-0) | **0.003** | -1.59 (-5-0) | | **0.03** | | | -3.54 (-7.54-1.28) | | | **0.01** |
| Δ TNFα EoT-w4 | -1.19 (-6.2-0) | 0.06 | -1.3 (-6.2-0) | | **0.05** | | | -0.9 (-1.6-0) | | | 0.11 |
| Δ TNFα SVR4-EoT | -0.63 (-0.7-0.5) | 0.64 | -0.48 (-0.64-0.5) | | 0.72 | | | -0 .62 (-0.7-0) | | | 0.55 |
| Δ TNFα SVR12-SVR4 | -0.1 (-1.8-0) | 0.74 | -0.26 (-0.3-0) | | 0.65 | | | -1.8 (-2.66-0.89) | | | **0.02** |
